# Quantifying the Contribution of Bone Morphology to Implant Selection in Shoulder Arthroplasty Using CT-Based Deep Learning

**DOI:** 10.3390/bioengineering13050574

**Published:** 2026-05-19

**Authors:** Andrea Moglia, Luca Marsilio, Matteo Rossi, Alfonso Manzotti, Luca Mainardi, Pietro Cerveri

**Affiliations:** 1 Department of Electronics, Information and Bioengineering, Politecnico di Milano, 20133 Milan, Italy; andrea.moglia@polimi.it (A.M.); matteo2.rossi@polimi.it (M.R.); luca.mainardi@polimi.it (L.M.); 2 Hospital ASST FBF-Sacco, 20154 Milan, Italy; alfonso.manzotti@asst-fbf-sacco.it; 3 Department of Industrial, and Information Engineering, University of Pavia, 27100 Pavia, Italy

**Keywords:** shoulder arthroplasty planning, deep learning, anatomical/reverse implant, clinical translation

## Abstract

We investigated whether bone morphology alone can inform implant selection in shoulder arthroplasty using a hypothesis-driven deep learning framework applied to preoperative computed tomography (CT) scans. The proposed approach extends a previously validated segmentation and pathology-staging pipeline by introducing implant-type prediction and a controlled human–AI comparison. The workflow combines CEL-UNet for 3D bone segmentation with ArthroNet^+^, a multi-task network assessing osteophytes, joint-space narrowing, humeroscapular alignment, and implant type. Trained on a multicenter cohort of 600 patients, CEL-UNet achieved Dice scores of 0.99 for the humerus and 0.98 for the scapula. ArthroNet^+^ achieved high performance in pathology classification (up to 95% for alignment tasks). Under morphology-only conditions, ten orthopedic surgeons achieved 61% accuracy with low inter-rater agreement (Fleiss’ κ≈0.15), while the model reached 78% agreement with the implant choices observed in the dataset, reflecting the ability to reproduce clinical decision patterns rather than to identify an optimal implant selection. This performance is characterized by a class-dependent asymmetry, with higher recall for reverse implants than for anatomical ones. These findings indicate that bone morphology provides a measurable but incomplete signal for implant selection, and should therefore not be interpreted as reflecting clinical decision-making performance. The framework quantifies the morphology-driven component of surgical decision making under controlled conditions, supporting future integration with multimodal clinical data.

## 1. Introduction

Osteoarthritis (OA) of the shoulder is a progressive degenerative joint condition characterized by cartilage loss, osteophyte formation, joint space narrowing, and bone remodeling. These changes frequently lead to pain, limited mobility, and functional impairment of the glenohumeral (GH) joint [1,2]. Surgical treatment, often involving shoulder arthroplasty, is indicated in moderate to severe cases. The main implant types include anatomical prostheses, which preserve natural biomechanics, and reverse prostheses, typically used when rotator cuff dysfunction is present or when joint alignment is compromised [3,4]. The demand for total shoulder arthroplasty (TSA) has increased markedly and is expected to continue rising. Between 2012 and 2017, the incidence of reverse TSA (rTSA) nearly tripled, from 7.3 to 19.3 cases per 100,000, while anatomical TSA (aTSA) increased from 9.5 to 12.5 cases per 100,000. Timely and accurate implant identification is critical, as delays can prolong operative time, increase costs, and heighten the risk of complications [5].

Preoperative evaluation of three key pathological features, osteophyte burden (OS), joint space narrowing (JS), and humeroscapular alignment (HSA), is central to assessing the overall condition of the shoulder joint and informing implant selection. Concentric alignment and preserved joint space often support anatomical implants, while eccentric alignment, joint collapse, and large osteophytes favor reverse prostheses [6,7]. However, reliable identification of these features remains challenging, particularly in cases with severe joint degeneration or large anatomical variability [8]. Recent developments in patient-specific instrumentation (PSI) have improved the accuracy and efficiency of shoulder arthroplasty planning [9,10,11,12]. These techniques rely on precise 3D reconstructions of the humerus and scapula derived from CT scans, making robust automated segmentation a critical component of the workflow.

Machine learning (ML) and deep learning (DL), particularly convolutional neural networks (CNNs), have demonstrated strong performance in medical image analysis [13,14]. Encoder–decoder architectures such as U-Net and its variants have been successfully applied to bone segmentation tasks [15,16,17]. Moreover, CNN-based approaches have shown promise in classifying osteoarthritic severity [18] and supporting treatment planning [19,20,21]. In shoulder arthroplasty, ML has been primarily used for predicting postoperative outcomes and functional recovery, with encouraging results in both small and large retrospective cohorts [22].

However, these approaches typically rely on multimodal inputs and do not isolate the contribution of individual information sources, such as bone morphology alone. Furthermore, relatively few studies have integrated segmentation, 3D reconstruction, and pathology staging within a unified pipeline specifically tailored to CT-based surgical planning.

In this work, we propose a unified deep learning framework for shoulder CT analysis that combines bone segmentation, anatomical reconstruction, pathology staging, and implant-type prediction. The proposed framework extends a previously validated segmentation and pathology-staging pipeline [18] by introducing implant-type prediction and a controlled human–AI comparison. The architecture is composed of two main components, namely CEL-UNet and ArthroNet^+^, and is trained and evaluated on a multicenter cohort of 600 patients. Importantly, the goal of this study is not to replicate clinical decision-making conditions, but to investigate whether implant selection can be inferred from osseous morphology alone under controlled input constraints. To this end, implant prediction is benchmarked against ten orthopedic surgeons who evaluate the same cases using only CT-derived 3D bone models, without access to additional clinical information. This experimental design enables a controlled comparison between human and algorithmic inference based exclusively on morphology, allowing us to quantify the extent to which implant selection decisions can be derived from bone structure alone. While such a setting does not reflect routine clinical practice, it provides a means to isolate the morphology-driven component of surgical decision making within a fundamentally multimodal process. The main contributions of this work are as follows:

1. a hypothesis-driven evaluation of CT-only implant selection. We assess whether osseous morphology alone contains sufficient information to differentiate anatomical from reverse shoulder implants under controlled, multimodal-data–limited conditions, explicitly isolating the contribution of bone structure within a fundamentally multimodal clinical decision process.
2. a fully automated multi-task pipeline for shoulder arthroplasty planning. The ArthroNet^+^ jointly predicts osteophyte severity, joint-space narrowing, humeroscapular alignment, and implant type from CT-derived glenohumeral regions, providing a unified framework for morphology-driven analysis while achieving real-time execution (≤15 s).
3. a blinded human–AI benchmark under controlled conditions. Using interactive 3D bone models, we compare model predictions with those of ten orthopedic surgeons operating under morphology-only conditions, thereby quantifying the morphology-driven component of surgical decision making and characterizing variability in expert interpretation under constrained input settings.

### Context and Clinical Rationale

**Figure 1:**
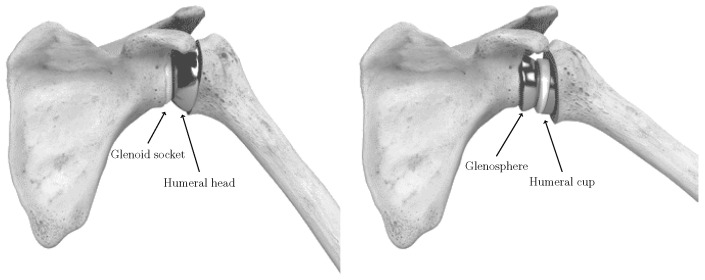
Anatomical and reverse implants in shoulder arthroplasty.

Shoulder arthroplasty requires careful preoperative assessment of glenohumeral joint degeneration and of the anatomical and functional factors that determine whether an anatomical or reverse prosthesis is most appropriate (Figure 1). In routine clinical practice, this decision is inherently multimodal: surgeons integrate information from radiographs, CT, MRI, patient age and functional demands, comorbidities, and particularly the integrity of the rotator cuff [23]. While cuff status is a dominant driver of implant selection, long-standing soft-tissue dysfunction often produces secondary, measurable alterations in bone morphology, including humeral head decentering, eccentric wear, joint-space collapse, and osteophyte proliferation [24,25]. These osseous changes are frequently evident on CT even when soft-tissue structures are not directly visualized [26]. Within this context, the present study is motivated by the hypothesis that specific morphological patterns of bone remodeling encode part of the information used in implant selection. We do not assume that CT-derived morphology provides a direct measure of rotator cuff integrity. Rather, we posit that chronic biomechanical imbalance leaves detectable structural footprints that can be captured and interpreted by a deep learning model. Accordingly, ArthroNet^+^ is designed to first infer clinically meaningful intermediate features, osteophyte severity, joint-space narrowing, and humeroscapular alignment, and then predict implant type based on these features, reflecting, in a simplified manner, the hierarchical reasoning process used in clinical practice. Importantly, the aim of this study is not to reproduce clinical decision-making conditions, but to isolate and quantify the contribution of osseous morphology under controlled input constraints. Importantly, implant labels correspond to observed surgical decisions and should not be interpreted as representing an optimal or consensus-based ground truth. To this end, both the model and the human evaluators are restricted to CT-derived bone information alone. While this setting does not reflect routine clinical practice, it enables a controlled investigation of the morphology-driven component of implant selection, disentangled from other clinical factors. The results should therefore be interpreted as an analysis of the information content of bone morphology, rather than as evidence that CT-only approaches can replace multimodal clinical evaluation. In this perspective, the proposed framework provides a tool to better understand the role of anatomical features in surgical decision making and to identify cases in which morphology alone may be insufficient, thereby supporting future integration with multimodal data in clinically realistic settings. The segmentation and pathology-staging components are reused from our previous work and are not modified in this study. They are included to enable a consistent end-to-end evaluation of the proposed framework.

## 2. Methodology

### 2.1. Clinical Data

The retrospective dataset, provided by MEDACTA International SA (Castel San Pietro, Switzerland), comprised axial CT scans of 600 patients in DICOM format. The dataset was multi-centric, encompassing North America (30%), Europe and the Middle East (48%), Asia Pacific (20%), and Latin America (2%). Between 2021 and 2022, all patients underwent shoulder arthroplasty using patient-specific instrumentation (PSI). Approximately 25% of patients received anatomical implants, while the remaining cases were treated with reverse implants. The patients had a mean age of 74 ± 11 years and were diagnosed mainly with osteoarthritis, inflammatory arthritis, osteonecrosis of the glenoid, and post-traumatic degenerative glenohumeral disease. The cohort showed a balanced gender distribution (50.4% male, 49.6% female). Although this information was available, demographic variables were not incorporated in the analysis in order to preserve the morphology-only experimental design. Due to data anonymization and data-sharing agreements, additional demographic variables (e.g., BMI, ethnicity) were not available. The CT scans featured mostly 512 × 512 pixels with an average of 330 slices, pixel sizes ranging from 0.30 to 0.98 mm, and slice thickness between 0.30 and 2.5 mm. The dataset included two distinct levels of annotation serving different purposes. First, the proximal humerus and scapula STL models were generated within the MEDACTA clinical workflow using semi-automated segmentation (Mimics v.16.0, Materialise) and manually reviewed by MEDACTA experts for PSI design. These segmentations were intended for surgical planning and guide production, rather than for diagnostic labeling. Second, for the purposes of the present study, additional diagnostic labels were defined and assigned independently. All diagnostic annotations were derived exclusively from preoperative CT images and the corresponding 3D reconstructed bone surfaces. No intraoperative or postoperative information was used. The labeling process was conducted by one musculoskeletal radiologist and subsequently reviewed by an expert orthopedic surgeon with over 20 years of experience. Three main clinical conditions were annotated, namely osteophyte severity (OS), joint-space condition (JS), and humeroscapular alignment (HSA), according to established radiological criteria:

- osteophyte severity (OS), three grades: <3 mm, 3–7 mm, >7 mm, based on the Samilson-Prieto grading system [8,27];
- joint space condition (JS), three grades: physiological, narrowed, non-detectable, based on the Kellgren–Lawrence grading system [1];
- humeroscapular alignment (HSA), two grades: concentric (physiological) vs. eccentric (pathological) [28].

OS and JS were defined as ordinal variables with three levels, while HSA was defined as a binary variable. For the purposes of model training, all labels were treated as independent categorical classes. Implant type labels corresponded to the surgical procedures performed in clinical practice and therefore reflected individual surgeon decisions rather than a consensus-based ground truth. Consequently, the dataset represented observed clinical decision patterns rather than an objectively validated optimal implant selection. As a consequence, the model learned historical clinical decision patterns rather than an objectively validated optimal implant selection.

### 2.2. Segmentation Task: CEL-UNet Model

**Figure 2:**
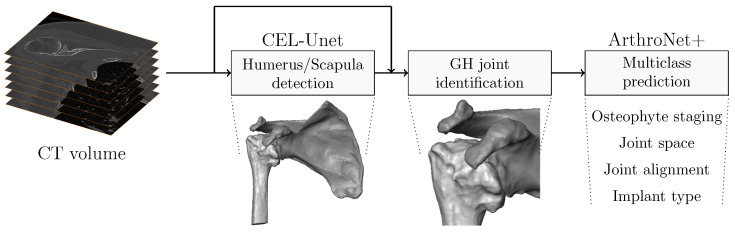
Pipeline schema of the proposed automatic methodology. In the I stage, the segmentation module CEL-UNet processes a CT scan to reconstruct humerus and scapula surfaces. The II stage identifies the glenohumeral region. The third stage, composed of a classification deep module, processed the GH joint region in the CT scan to detect three different clinical conditions and predict anatomical against reverse implant.

The developed processing pipeline was based on a cascade of two deep learning networks, CEL-UNet for shoulder bone segmentation and ArthroNet^+^ for diagnostics and implant-type prediction (Figure 2). The framework builds upon a previously validated deep learning pipeline for shoulder CT analysis [18], which included CEL-UNet for bone segmentation and ArthroNet for pathology staging. In the present study, these components are reused without architectural modifications, and previously reported segmentation performance is summarized to provide context for the overall pipeline. The main extension introduced here consists of (i) the addition of an implant-type prediction branch within the multi-task architecture (ArthroNet^+^), and (ii) a controlled human–AI comparison under morphology-only conditions. Accordingly, the novelty of this work lies primarily in the implant prediction task and in the experimental evaluation protocol, rather than in modifications of the underlying segmentation architecture. Accordingly, the segmentation component is not a novel contribution of the present study and is reported only to provide context for the overall pipeline. The CEL-UNet, extending the standard U-Net architecture, featured an encoder path with skip connections directed to two parallel decoder branches: the upper branch segments regions of interest, while the lower branch focuses on edge detection using a pyramidal, multi-scale extraction strategy. To enhance segmentation accuracy, edge features from each layer of the edge decoder were fused into the corresponding layers of the segmentation decoder, enabling edge-guided refinement of region predictions. CEL-UNet incorporates a multi-scale edge extraction module that uses a pyramidal structure to aggregate features across different resolutions, allowing the network to accurately capture fine edge details even after spatial down-sampling. To further enhance segmentation, unidirectional skip connections were introduced from each block of the edge detection decoder to the corresponding block in the region segmentation decoder, enabling effective fusion of edge and region features at every decoding stage. This architecture was previously shown to improve bone boundary detection in osteoarthritic anatomy [16,18].

This architecture was specifically motivated by the challenges of osteoarthritic bone CT segmentation, where osteophytes, cortical irregularities, and joint-space collapse produce highly fragmented and irregular bone boundaries. In such scenarios, accurate delineation of fine contours is essential for reliable 3D reconstruction, yet state-of-the-art segmentation frameworks such as nnU-Net do not explicitly include edge-focused learning mechanisms. As far as training is concerned, each decoding path was characterized by its own specialized loss function, namely the region ($L_{r}$) and edge ($L_{e}$) segmentation losses. $L_{r}$ combines a class-weighted Dice score (D) and a distance-weighted cross-entropy term (C). D was computed independently for each class and weighted by the class frequency $k_{c}$ to address imbalance between humerus (c=1) and scapula (c=2). The region segmentation loss was defined as:

(1) $$L_{r}=1-\left(\alpha \cdot D+\left(1-\alpha \right)\cdot C\right)$$

where α balances the two contributions. The cross-entropy term incorporates spatial weighting through a distance-weighted map (*DWM*), derived from the Euclidean distance transform (*EDT*), defined as:

DWM=exp(−EDT)

This weighting scheme increases the importance of boundary voxels during training. Accordingly, the cross-entropy loss is computed as:

(2) $$C\left(y,\hat{y}\right)=-\sum_{c=1}^{C}k_{c}\left(\sum_{i=1}^{N}DWM_{c}\cdot y_{c}\cdot \log\left({\hat{y}}_{c}\right)\right)$$

where $y_{c}$ and ${\hat{y}}_{c}$ denote the ground truth and predicted segmentation for class *c*, respectively. The edge loss $L_{e}$ is defined as:

(3) $$L_{e}=1-\left(\beta \cdot C+\left(1-\beta \right)\cdot \hat{C}\right)$$

where $\hat{C}$ is the reverse cross-entropy:

(4) $$\hat{C}\left(y,\hat{y}\right)=-\sum_{c=1}^{C}k_{c}\left(\sum_{i=1}^{N}DWM\cdot \left(1-y_{c}\right)\cdot \log\left(1-{\hat{y}}_{c}\right)\right)$$

This dual-loss formulation encourages the model to prioritize both region accuracy and boundary precision. The use of distance-weighted maps has been shown to enhance performance in anatomically complex regions, particularly in the presence of osteoarthritic remodeling [16,18].

### 2.3. Diagnostics and Implant Type Prediction: The ArthroNet^+^ Model

**Figure 3:**
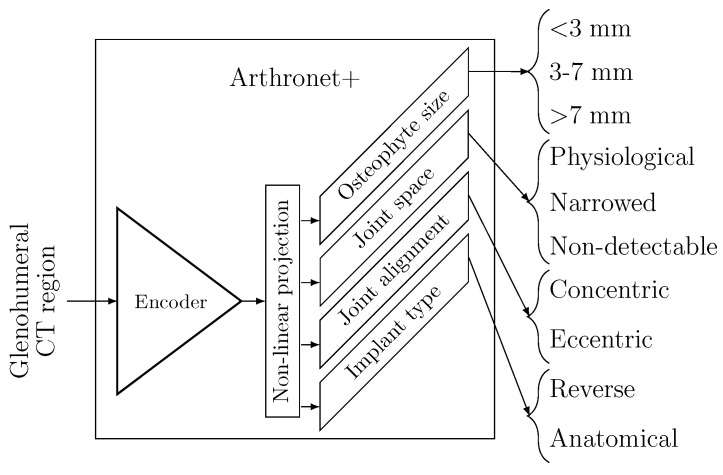
ArthroNet^+^ model. It incorporates a front-end encoder, which processes 3D CT images of the glenohumeral joint, a non-linear projection layer, and four task-dependent Softmax layers to predict osteophyte (3 labels), joint space (3 labels), joint alignment (2 labels), along with implant type (2 labels).

Following segmentation, the proximal humerus and scapula bones were digitally reconstructed using the traditional marching cubes algorithm. The glenohumeral joint region was then automatically isolated from the CT scans, serving as the input for the ArthroNet^+^ model (Figure 3). The ArthroNet^+^ model extends the previously introduced ArthroNet architecture [18] by incorporating an additional task-specific branch for implant-type prediction within the same multi-task framework. It is a multi-task deep model designed to jointly classify the three clinically relevant conditions of the GH joint and predict the implant type (anatomical vs. reverse). As anticipated above, the model operated on a subregion of the original CT scan embedding the GH joint. The architecture consisted of a deep convolutional encoder followed by a non-linear projection layer mapping the condensed features into a shared latent space optimized for multi-class separation. The final part of the network included four parallel task-specific branches, each ending with a dedicated Softmax output layer. This design reflects, in a simplified manner, the combination of morphological cues typically considered during clinical assessment. By learning these correlated tasks jointly, ArthroNet^+^ leveraged shared representations of GH morphology and improved robustness of the implant-type inference. Since anatomical implants represented approximately 25% of the cohort and reverse implants the remaining 75%, the implant-type prediction task was affected by a substantial class imbalance. To mitigate this effect, we employed a class-weighted cross-entropy loss, where each class weight was defined to be inversely proportional to its frequency in the training set. Specifically, for each class *c*, the weight $w_{c}$ was computed as:

(5) $$w_{c}=\frac{\frac{1}{N_{c}}}{\sum_{i=1}^{C}\frac{1}{N_{i}}},$$

where $N_{c}$ denotes the number of samples belonging to class *c* and *C* is the total number of classes. This formulation ensured that minority classes contributed proportionally more to the loss during optimization, thereby reducing prediction bias toward the majority reverse-implant class. No oversampling or synthetic augmentation of anatomical cases was applied, in order to avoid potential overfitting and to preserve the natural data distribution. The source code of the proposed framework is publicly available (Available online: https://github.com/LucaMarsilio/AI-Shoulder (accessed on 11 April 2026)).

### 2.4. Training the Models

Training and testing procedures for both deep models were performed on a 32-core CPU and NVIDIA A100-PCIE GPU with 40 GB RAM. The dataset (600 cases) was split into a training (500) and a test (100) set. The distribution of OS, JS, and HSA categories across the training and test sets showed a moderate imbalance. In the test set, OS classes included 26, 33, and 41 cases, JS classes 33, 28, and 39 cases, and HSA classes 63 and 37 cases. The test set was balanced between anatomical (50) and reverse (50) implant types, as determined from surgical records. This balanced distribution was intentionally adopted to enable a controlled evaluation of class-wise performance and to avoid bias due to class imbalance, rather than to reflect real clinical prevalence. Early stopping was used to prevent overfitting. As an example for CEL-Unet and ArthroNet^+^ models, one training took 98 and 183 epochs with an overall training time of about 78 h and 5 h, respectively. Considering the overall pipeline at inference time (CT segmentation, 3D bone reconstruction, GH joint detection in the CT scan, and prediction), the process took on average less than 15 s. This confirmed a high-speed throughput, highlighting the efficiency of the proposed cascaded multi-task approach, making it potentially suitable for clinical practice.

### 2.5. Testing Protocols

#### 2.5.1. Evaluation Metrics

The performance of the CEL-UNet model in segmenting the humerus and scapula on the test set was rigorously evaluated by comparing its results against the state-of-the-art nnUNet [13]. The nnUNet was trained using two distinct loss functions: distance cross-entropy (DCE) and focal (FOC) losses. The primary metrics employed for this evaluation were:

- Dice Index: This metric assesses the spatial overlap between the segmented bone regions and the ground truth. A higher Dice index indicates better segmentation accuracy.
- Jaccard Index: also known as the Intersection Over Union (IOU), the Jaccard index quantifies the similarity and diversity of the segmented and ground truth sets. Like the Dice index, a higher value signifies superior segmentation.

Along with Dice and IOU indexes, precision and recall were computed. To quantitatively evaluate the ArthroNet^+^ performance, the accuracy was computed along with two commonly adopted metrics as the Macro F1-score and Weighted F1-score. Both metrics derive from the F1-score, which classically defines the harmonic mean of precision and recall for each class. However, they differ in the way they aggregate class-wise performance. Macro F1-score computes the F1-score independently for each class and then averages them, giving equal weight to each class, regardless of its prevalence in the dataset. It is defined as:

(6) $$\mathrm{Macro}\;F1=\frac{1}{N}\sum_{i=1}^{N}\frac{2\cdot {\mathrm{Precision}}_{i}\cdot {\mathrm{Recall}}_{i}}{{\mathrm{Precision}}_{i}+{\mathrm{Recall}}_{i}}$$

where *N* is the total number of classes (e.g., humerus and scapula), and ${\mathrm{Precision}}_{i}$, ${\mathrm{Recall}}_{i}$ refer to the precision and recall for class *i*, recalling that, for sake of clarity, 0, 1 and 2 are the background, the humerus and the scapula, respectively.

Weighted F1-score incorporates the support of each class, namely the number of true instances for that class, and weights the F1-score accordingly. This approach accounts for class imbalance and better reflects overall segmentation performance in realistic, heterogeneous datasets. The metric is defined as:

(7) $$\mathrm{Weighted}\;F1=\frac{1}{\sum_{i=1}^{N}s_{i}}\sum_{i=1}^{N}s_{i}\cdot \frac{2\cdot {\mathrm{Precision}}_{i}\cdot {\mathrm{Recall}}_{i}}{{\mathrm{Precision}}_{i}+{\mathrm{Recall}}_{i}}$$

where $s_{i}$ is the support of class *i*, defined as the number of voxels belonging to that class in the ground truth segmentation.

In order to verify potential intra-class bias, especially for OS and JS, the label-dependent recall and precision metrics were also compared.

#### 2.5.2. Blinded Surgeon Evaluation Under Morphology-Only Conditions

To assess the extent to which implant selection can be inferred from osseous morphology alone, a blinded evaluation was conducted involving a panel of 10 orthopedic surgeons. All participating testers were affiliated with a single institution, ASST Fatebenefratelli–Sacco Hospital (Milan, Italy), ensuring a consistent clinical background while still representing a range of experience levels. This choice was intended to reduce variability due to differing institutional protocols, although individual decision-making strategies may still vary. The 100 cases used for the blinded surgeon evaluation corresponded exactly to the held-out test set defined in Section 2.4. For each case, only the 3D-reconstructed surfaces of the humerus and scapula were provided. These surfaces were generated from preoperative CT scans using the CEL-UNet model. All non-osseous information was intentionally removed, and no clinical, radiological, or patient metadata (e.g., age, diagnosis, rotator cuff status) was disclosed to the evaluators.

This experimental setup was designed to enforce morphology-only conditions for both the surgeons and the deep learning model, thereby isolating the contribution of bone morphology to implant selection. Although both evaluations rely exclusively on morphology-derived information, the model operates on CT-derived volumetric data, whereas surgeons evaluate 3D reconstructed bone surfaces, reflecting different representations of the same anatomical structures. It is important to emphasize that this setting does not reflect routine clinical practice, in which surgeons rely on multimodal information. Instead, it represents a controlled scenario aimed at quantifying morphology-driven decision signals. The 3D reconstructions were evaluated using a custom graphical interface that allowed full interactive inspection, including free rotation, zooming, and visualization of individual or combined bone structures. This ensured that evaluators could analyze anatomical features without restrictions on viewpoint. The collected responses were compared against the implant types performed in clinical practice, which were used as reference labels. These labels represent surgical decisions rather than an objective ground truth and may reflect surgeon-specific preferences. This difference in data representation is acknowledged as a limitation of the experimental design and is considered in the interpretation of the results. Inter-rater agreement among surgeons was quantified using Fleiss’ κ, which measures the degree of agreement beyond chance for categorical ratings. The statistics is defined as:

(8) $$\kappa =\frac{P_{o}-P_{e}}{1-P_{e}}$$

where $P_{o}$ denotes the observed agreement and $P_{e}$ the agreement expected by chance. The observed agreement is computed as:

(9) $$P_{o,i}=\frac{1}{k(k-1)}\sum_{j=1}^{C}n_{ij}\left(n_{ij}-1\right)$$

(10) $$P_{o}=\frac{1}{N}\sum_{i=1}^{N}P_{o,i}$$

and the expected agreement as:

(11) $$P_{j}=\frac{1}{Nk}\sum_{i=1}^{N}n_{ij}$$

(12) $$P_{e}=\sum_{j=1}^{C}P_{j}^{2}$$

In this study, κ was used to quantify the consistency of morphology-based decisions under constrained input conditions, rather than to assess agreement in a clinical setting. Low values of κ were therefore interpreted as reflecting variability in morphology-driven assessments, which may arise either from anatomical ambiguity or from differences in individual decision criteria among surgeons. To further characterize case-wise disagreement, the entropy of surgeon predictions was computed. Let $p_{1}$ and $p_{2}$ denote the proportion of surgeons predicting anatomical (c=1) and reverse (c=2) implants, respectively. The entropy *H* was defined as:

(13) $$H=-\sum_{c=1}^{2}p_{c}\log_{2}\left(p_{c}\right)$$

with:

(14) $$p_{1}=\frac{n_{1}}{N},\;p_{2}=\frac{n_{2}}{N}$$

where $n_{1}$ and $n_{2}$ represent the number of surgeons selecting anatomical and reverse implants, respectively, and *N* is the total number of surgeons. The entropy ranges from 0 (complete agreement) to 1 (maximum disagreement). In this context, entropy served as a surrogate measure of variability in morphology-based decisions under constrained conditions. High entropy indicated disagreement among surgeons when only bone morphology is available, while low entropy reflects consistent interpretation of anatomical features. Finally, to estimate the predictive signal contained in collective human judgment, a logistic regression model was trained using the binary predictions of the ten surgeons as input features to predict the implant type. This “virtual consensus” approach provides an aggregate estimate of morphology-based decision patterns without assuming that individual or collective decisions represent an absolute ground truth.

## 3. Results

### 3.1. CEL-Unet: Segmentation Results

Focusing on the humerus (Table 1), Dice and Jaccard distributions achieved with the CEL-UNet were statistically higher than those of the two nnUNet variants (DCE and FOC). CEL-UNet also outperformed DCE-nnUNet in terms of precision and surpassed FOC-nnUNet in recall, confirming its robustness in capturing both the volumetric extent and detailed boundaries of the humeral structure. Regarding the scapula, CEL-UNet demonstrated statistically significant superiority (*p* = 0.01) over both baseline networks for Dice (humerus: *p* = 0.0002, scapula: *p* = 0.0005), Jaccard (humerus: *p* = 0.0005, scapula: *p* = 0.0008), and precision (humerus: *p* = 0.0002, scapula: *p* = 0.0002) scores, with a notably high precision (0.99) and a narrow interquartile range, indicating highly consistent performance. Although DCE-nnUNet achieved the highest recall for the scapula (0.97), CEL-UNet maintained competitive recall (0.96) while offering superior balance across all metrics. These results highlighted the advantages of edge-guided learning in complex anatomical regions, particularly when segmenting bones affected by osteoarthritic remodeling.

### 3.2. ArthroNet^+^: Diagnostic and Implant Type Prediction

**Figure 4:**
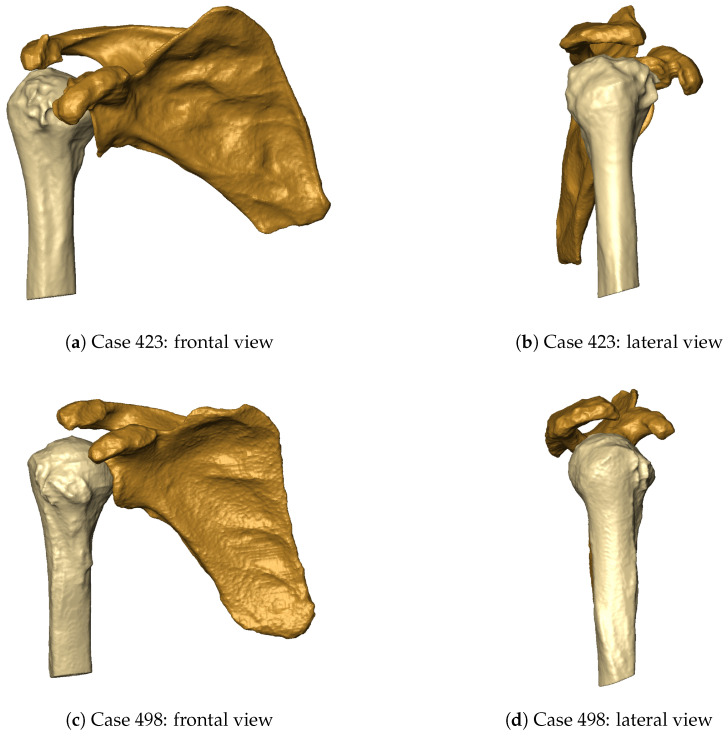
Representative cases of misclassification for osteophyte severity (OS) and joint space (JS). Both cases exhibit borderline morphological patterns, illustrating the difficulty of classifying intermediate severity grades.

The classification performance of the ArthroNet^+^ model across the four prediction tasks is summarized in Table 2. For the OS task, the model achieved an accuracy of 0.72, with comparable Macro F1-score and Weighted F1-score (0.73 and 0.72, respectively), reflecting moderate but balanced performance across classes. In the JS task, performance improved, reaching an accuracy of 0.81 with consistent precision and recall values (both around 0.80), indicating a reliable classification of joint-space conditions. The HSA task achieved the highest performance, with an accuracy of 0.95 and consistently high precision and recall (0.96 and 0.93), confirming the strong geometric separability of this feature. Finally, implant type (IT) prediction reached an accuracy of 0.78, with balanced Macro F1 and Weighted F1 scores (both 0.78). A deeper analysis of per-class metrics (Table 3) shows that the model exhibits asymmetric performance across implant classes. In particular, reverse implants (class 0) are identified with high recall (0.92) but moderate precision (0.71), indicating a tendency toward overprediction of this class. Conversely, anatomical implants (class 1) show higher precision (0.88) but lower recall (0.64), suggesting that a subset of anatomical cases is misclassified as reverse. For the OS and JS tasks, intermediate severity grades remain the most challenging to classify, consistent with the presence of transitional morphological patterns. In both tasks, mild conditions tend to be detected with higher recall than precision, indicating slight overestimation, whereas more severe cases are identified with higher precision, reflecting clearer structural signatures. Cases with eccentric glenoid wear or borderline joint-space narrowing accounted for most disagreements, in line with prior observations [29]. Figure 4d illustrates representative cases where both OS and JS are misclassified, highlighting the difficulty of interpreting borderline morphological patterns. For HSA, classification results were well balanced, with precision and recall both above 0.90. In contrast, performance for the IT task was asymmetric: the model showed high recall for reverse implants (0.92) but lower precision (0.71), indicating a tendency to overpredict this class. Conversely, anatomical implants were identified with higher precision (0.88) but lower recall (0.64), suggesting that a subset of anatomical cases was misclassified as reverse. This imbalance reflects the greater morphological distinctiveness of reverse cases compared to anatomically borderline conditions.

### 3.3. Surgeon Cohort: Implant Type Prediction Task

**Figure 5:**
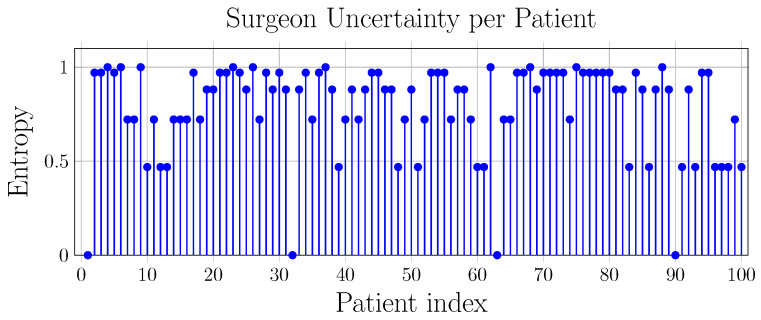
Entropy per patient. H=0 and H=1 indicate perfect agreement and maximum disagreement among surgeons. High-entropy cases are generally associated with borderline morphological patterns, such as intermediate osteophyte severity or partial joint-space narrowing, which may contribute to variability in morphology-based interpretation.

**Figure 6:**
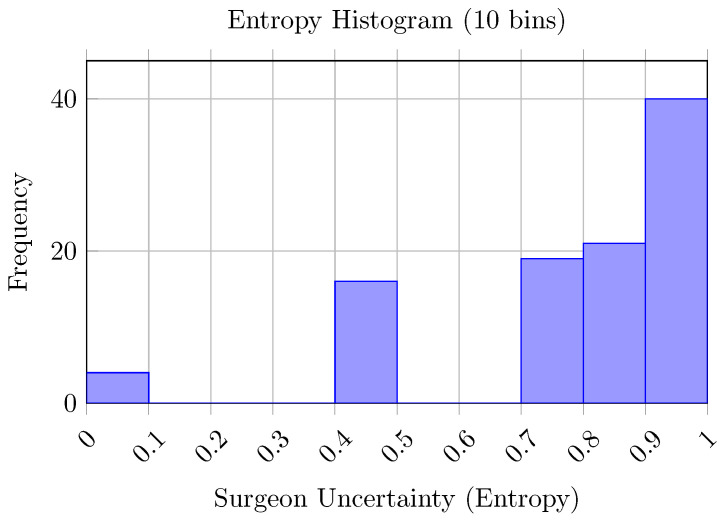
Distribution of entropy values across cases, reflecting variability in morphology-based implant selection.

**Figure 7:**
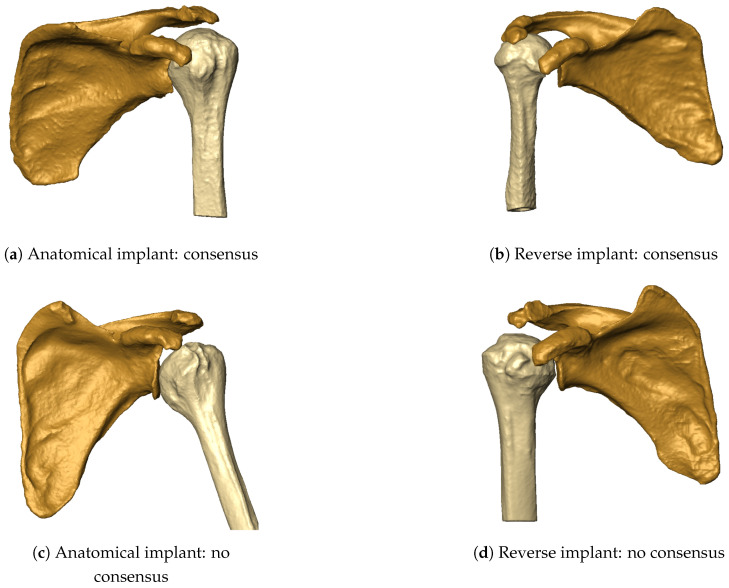
Examples of cases with full agreement (H=0) and maximal disagreement (H=1) among surgeons, highlighting the impact of morphological ambiguity on implant-type selection.

The overall accuracy of implant type prediction ranged from 51% to 69% across the surgeon cohort (Table 4), with a mean accuracy of 0.61 (SD: 0.06). Reverse implant sensitivity (mean: 0.69, SD: 0.09) was generally higher than anatomical implant sensitivity (mean: 0.53, SD: 0.07). Across most evaluators, anatomical false negatives were more frequent than reverse false negatives, indicating a tendency to classify anatomical cases as reverse under morphology-only conditions. Cases associated with high disagreement typically exhibited borderline morphological features, including moderate osteophyte formation, partial joint-space narrowing, or mild humeral head decentering. These patterns are consistent with intermediate severity grades, where structural alterations are less pronounced and more difficult to interpret in the absence of additional clinical information. Variability in performance was observed across surgeons with different experience levels, although no consistent relationship between experience and accuracy could be identified. The computed Fleiss’ κ was approximately 0.15, indicating low agreement among surgeons. This variability was further characterized through the entropy distribution of case-wise predictions (Figure 5 and Figure 6). Full agreement (H=0) was observed in four cases, while maximal disagreement (H=1) occurred in ten cases, reflecting substantial variability in morphology-based assessments. Selected examples of cases with full agreement and maximal disagreement are shown in Figure 7.

Despite this variability, the logistic regression model trained on aggregated surgeon predictions achieved an accuracy of 67%, indicating that collective morphology-based assessments contain a measurable predictive signal, although not sufficient to reach consistently high accuracy under these constrained conditions. Table 5 summarizes implant-type prediction performance under different input conditions. The “Original Surgeons” row is reported for reference only, as it corresponds to the surgical decisions used as ground truth labels and does not represent an independent evaluation benchmark. It should be noted that, although both evaluations are restricted to morphology-derived information, the model and surgeons operate on different data representations, as discussed in Section 2.5.2. When restricted to CT-derived bone morphology, surgeons achieved an average accuracy of approximately 61%, with lower sensitivity for anatomical implants compared to reverse implants. Under the same input constraints, ArthroNet^+^ achieved an accuracy of approximately 78%, with higher sensitivity for reverse implants compared to anatomical ones. Overall, ArthroNet^+^ predictions were consistent with the majority surgeon vote in 65% of cases. A non-parametric McNemar’s test (p=0.0003) indicated a statistically significant difference between model predictions and surgeon responses under these constrained conditions. These results quantify differences in morphology-based classification performance without reflecting clinical decision-making conditions.

## 4. Discussion

### 4.1. Main Findings

This study investigated whether information relevant to shoulder arthroplasty planning can be extracted from CT-derived bone morphology alone within a controlled experimental setting. The proposed pipeline integrated segmentation, anatomical reconstruction, pathology staging, and implant-type prediction, enabling a systematic analysis of morphology-driven inference. The first key result concerns the segmentation backbone. The CEL-UNet module achieved near-ceiling agreement with expert-verified labels for both the humerus and scapula, outperforming strong nnU-Net baselines across overlap metrics (Table 1). Beyond segmentation accuracy, this result supports the role of edge-aware learning in osteoarthritic anatomy, where cortical irregularities and osteophytes challenge boundary delineation. The resulting high-fidelity 3D reconstructions provide stable inputs for downstream analysis and reduce error propagation within the pipeline. Building on this anatomical representation, ArthroNet^+^ addressed four classification tasks: osteophyte severity, joint space condition, humeroscapular alignment, and implant type. Performance varied across tasks, with humeroscapular alignment showing the highest reliability, consistent with its strong geometric signature, while osteophyte severity and joint space classification showed moderate accuracy (Table 3). Errors were primarily concentrated in intermediate severity grades, where morphological overlap and transitional features are expected. Implant-type prediction achieved consistent agreement with the implant choices observed in the dataset, but exhibited class asymmetry, with higher sensitivity for reverse implants compared to anatomical ones (Table 2). This agreement should be interpreted as the model’s ability to reproduce observed clinical decision patterns, rather than as evidence of optimal or prescriptive implant selection. Analysis of misclassified anatomical cases revealed that most errors occurred in borderline morphologies, where osseous changes were subtle and implant selection is likely driven by soft-tissue factors not encoded in bone structure (Figure 7). These findings indicate that morphology-based inference is most reliable when structural alterations are pronounced, and becomes less informative when soft-tissue pathology dominates. Consistent with these observations, the blinded surgeon evaluation under morphology-only conditions revealed substantial inter-rater variability and a tendency to favor reverse implants. Although the surgeon cohort included different levels of clinical expertise, its limited size (n = 10) did not allow for statistically robust inference on human performance, and the observed variability should therefore be interpreted as a qualitative indicator of morphology-based uncertainty under constrained conditions. This variability reflects the difficulty of performing implant selection based solely on bone morphology and highlights the limitations of morphology-only reasoning. Under the same input constraints, the model produced more consistent predictions, reflecting its deterministic nature rather than an inherently superior clinical decision process. Entropy-based analysis further identified cases with high disagreement among surgeons, indicating variability in morphology-based interpretation under constrained conditions (Figure 5). These cases likely correspond to situations in which bone morphology alone does not provide sufficient information to support a clear implant selection, reinforcing the need for multimodal assessment in clinical practice.

### 4.2. Comparison with Literature Papers

Comparable efforts in applying AI to shoulder arthroplasty have primarily addressed implant sizing, outcome prediction, or radiographic implant recognition rather than the CT-only decision between anatomical and reverse prostheses (Table 6). Machine learning-based clinical decision support tools for shoulder arthroplasty were proposed, focusing on predicting postoperative outcomes from multimodal clinical data [30]. These approaches differ from the present study, which aims to isolate the contribution of CT-derived bone morphology rather than optimize clinical decision-making performance. In a multi-surgeon study explicitly addressing surgical type selection, an ML tool matched surgeons’ recommendations on anatomical vs reverse shoulder implant in 78% of cases, indicating moderate agreement but leaving room for improvement in implant-type decision support [31]. By contrast, CT-based digital templating consistently achieves high concordance for component sizing (within one size in 99% for glenoid and 88–98% for humeral components), and a recent comparative analysis found no significant advantage of complex ML over simpler linear models for predicting implant sizes from demographics [24]. These approaches addressed component sizing rather than implant-type selection and therefore do not directly inform the decision between anatomical and reverse prostheses. Related radiographic AI tasks, identifying implant type or manufacturer, reported accuracies around 94–97%, again adjacent to but not directly addressing preoperative implant-type selection [32]. However, these methods focus on postoperative recognition rather than preoperative decision making, and therefore address a fundamentally different clinical task. Within this context, our CT-only framework addresses the specific problem of implant-type inference from osseous morphology under controlled input conditions. Rather than directly comparing clinical performance, the present study focuses on quantifying the information content of bone morphology and its relationship to observed surgical decisions. Compared with our present work, which focuses on extracting decision-relevant information from osseous morphology, in [20] the authors showed that deep learning can also quantify rotator cuff muscle atrophy and fatty infiltration directly from routine CT with expert-level accuracy. While their model targeted soft-tissue degeneration and ours predicted implant type from bone-only features, the two approaches are complementary and show that CT encodes multiple dimensions of shoulder pathology relevant for arthroplasty planning. Their findings also suggest that integrating CT-based muscle degeneration metrics into our framework could reduce ambiguity in anatomical implant classification, particularly when cuff dysfunction rather than bone remodeling drives the surgical decision. Multitask radiograph-based model was proposed to segment images and classify benign-versus-malignant primary bone tumors [33], achieving about 80% diagnostic accuracy. Although addressing a different clinical application, their results align with ours in showing that multitask deep learning can enhance diagnostic performance and outperform less-experienced clinicians while approaching expert-level accuracy.

### 4.3. Clinical Implications

The findings of this study should be interpreted within the context of a controlled experimental design aimed at isolating the contribution of bone morphology to implant selection. As such, the proposed framework is not intended to replace clinical decision making, but rather to provide insight into how morphological features relate to observed surgical choices. From a clinical perspective, the results highlight that osseous morphology alone contains a measurable but incomplete decision signal. In cases where bone remodeling is pronounced, morphology may provide sufficient information to support consistent interpretation. However, in cases with subtle structural changes, implant selection appears to depend predominantly on soft-tissue factors and patient-specific clinical variables that are not captured by CT. The observed variability among surgeons under morphology-only conditions further supports the notion that implant selection cannot be reliably performed without multimodal information. In this context, the proposed model can be interpreted as a tool for analyzing morphology-driven patterns rather than as a decision-support system in routine clinical practice. Potential applications of this framework may therefore lie in research and educational settings, where it can be used to study the relationship between bone morphology and surgical decisions, or to identify cases in which morphological information is insufficient and additional imaging or clinical evaluation is required.

### 4.4. Work Limitations

Despite the promising results obtained in this study, some limitations must be acknowledged. First, the model was intentionally designed to operate exclusively on CT-derived osseous morphology in order to test whether bone structure alone embeds sufficient information to support implant selection. However, clinical decision making in shoulder arthroplasty is inherently multimodal. Surgeons routinely integrate factors that are not visible on CT, including rotator cuff integrity, patient age and functional expectations, bone quality, comorbidities, and overall functional status. These determinants frequently outweigh morphology alone, particularly in anatomical implant selection. Accordingly, the present work should be interpreted as quantifying the morphology-driven component of surgical decision making, rather than suggesting that CT alone is sufficient for definitive implant selection. Second, implant type labels used in this study correspond to surgical decisions performed in clinical practice and therefore reflect individual surgeon judgment rather than a consensus-based or objectively validated ground truth. As a result, the model learns to reproduce observed clinical decision patterns, which may include surgeon-specific preferences and variability. Third, although CT-only inference may be informative in specific analytical contexts, such as understanding morphology-driven patterns or identifying cases with ambiguous structural features, it does not reflect routine clinical conditions. In practice, implant selection is performed using multimodal information, and the constrained experimental setup adopted in this study should not be interpreted as representative of clinical workflows. Fourth, despite the multicentric nature of the dataset, the distribution of implant types was imbalanced, with anatomical implants underrepresented, reflecting current epidemiological trends. Moreover, the use of a balanced test set, while suitable for controlled analysis, does not reflect the true prevalence of implant types in clinical practice and may therefore influence the reported accuracy. Although the dataset is multicentric and the test set was randomly sampled across centers, this does not constitute external validation, and further evaluation on independent datasets is required to assess generalizability across institutions and imaging protocols. Although class weighting during training mitigated this imbalance, future work should aim to include more balanced cohorts and incorporate additional clinical variables. Finally, the comparison with surgeons was conducted under deliberately constrained CT-only conditions to ensure a controlled evaluation aligned with the model input. While this design enables isolation of morphology-driven information, it limits the direct applicability of the results to clinical decision making. Future investigations should focus on integrating multimodal data and evaluating model performance in settings that more closely reflect real clinical practice.

## 5. Conclusions

This study introduced a fully automated deep learning pipeline for the analysis of shoulder CT data, integrating segmentation, pathology staging, and implant-type inference within a unified framework. The model was trained on implant choices observed in clinical practice, enabling it to capture associations between osseous morphology and surgical decision patterns. In this perspective, the model should be viewed as a tool for analyzing morphology-driven decision signals rather than for guiding clinical decision making. When evaluated under controlled morphology-only conditions, both the model and the surgeon cohort exhibited limitations in implant-type prediction, reflecting the reduced information content available when multimodal clinical data are not accessible. The observed variability among surgeons and the performance of the model indicate that bone morphology alone contains a measurable but incomplete signal for implant selection. These findings should be interpreted as an analysis of the morphology-driven component of surgical decision making, rather than as evidence that CT-only approaches can support clinical decision making. In particular, the results highlight that morphology-based inference is most informative when structural alterations are pronounced, while it becomes less reliable in cases where implant selection depends primarily on soft-tissue factors. Overall, this study provides a framework for quantifying the contribution of anatomical features to surgical decisions and for identifying cases in which morphology alone is insufficient. Future work should focus on integrating multimodal clinical information and evaluating model performance in settings that more closely reflect real-world clinical practice.

## Figures and Tables

**Table 1:** Dice, Jaccard, precision, and recall median and IQR scores for humerus (above) and scapula (below) comparing the performances of the three networks (CEL-UNet, and DCE- and FOC-nnUNet) over the independent test set of 100 cases. The asterisk symbol (*) defines a statistically significant difference (*p* = 0.01) between the outcome distributions against the two other networks.

	Humerus			
	Dice	Jaccard	Precision	Recall
DCE-nnUNet	0.98 (0.97–0.99)	0.97 (0.94–0.98)	0.98 (0.97–0.99)	0.98 (0.97–0.99)
FOC-nnUNet	0.98 (0.97–0.99)	0.96 (0.94–0.98)	0.99 (0.98–0.99)	0.97 (0.96–0.98)
CEL-UNet	0.99 * (0.98–0.99)	0.98 * (0.97–0.99)	0.99 (0.98–0.99)	0.99 (0.98–0.99)
	**Scapula**			
	**Dice**	**Jaccard**	**Precision**	**Recall**
DCE-nnUNet	0.97 (0.96–0.98)	0.95 (0.92–0.96)	0.97 (0.95–0.98)	0.97 * (0.96–0.98)
FOC-nnUNet	0.97 (0.96–0.98)	0.94 (0.92–0.96)	0.97 (0.96–0.98)	0.97 (0.95–0.98)
CEL-UNet	0.98 * (0.97–0.98)	0.95 * (0.94–0.96)	0.99 * (0.99–0.99)	0.96 (0.95–0.97)

**Table 2:** Classification performance of ArthroNet^+^ on the test set across the four prediction tasks: osteophyte severity (OS), joint space (JS), humeroscapular alignment (HSA), and implant type (IT).

Task	Accuracy	Precision	Recall	Macro F1	Weighted F1
OS	0.72	0.74	0.75	0.73	0.72
JS	0.81	0.81	0.80	0.80	0.81
HSA	0.95	0.96	0.93	0.94	0.95
IT	0.78	0.80	0.78	0.78	0.78

**Table 3:** Per-class Classification metrics per task. Accuracy, Precision, Recall, and F1-score are reported for each class, along with overall task accuracy.

Task	Class	Precision	Recall	F1-Score
OS	0	0.80	0.92	0.85
	1	0.58	0.75	0.66
	2	0.85	0.56	0.68
		Accuracy = 0.72		
JS	0	0.78	0.99	0.88
	1	0.69	0.64	0.66
	2	0.93	0.76	0.85
		Accuracy = 0.81		
HSA	0	0.93	0.98	0.95
	1	0.98	0.86	0.92
		Accuracy = 0.95		
IT	0	0.71	0.92	0.80
	1	0.88	0.64	0.74
		Accuracy = 0.78		

**Table 4:** Implant type selection over 50 anatomical and 50 reverse cases. Ten orthopedic surgeons participated in the evaluation. A SE: anatomical implant sensitivity; R SE: reverse implant sensitivity; A TP: anatomical implant true positives; R TP: reverse implant true positives; A FN: anatomical implant false negatives; R FN: reverse implant false negatives. CoD: Chief of Department (>25 years), Con: Consultant (≈10–15 years), Reg: Registrar (≈5–10 years), Res: Resident (≈2–4 years).

ID	Role	Accuracy	A SE	R SE	A TP	R TP	A FN	R FN
01	CoD	0.65	0.58	0.72	29	36	21	14
02	Con	0.62	0.51	0.73	26	36	25	13
03	Res	0.62	0.53	0.71	27	35	24	14
04	Con	0.55	0.47	0.63	24	31	27	18
05	Con	0.69	0.64	0.74	32	37	18	13
06	Reg	0.51	0.48	0.54	24	27	26	23
07	Reg	0.60	0.60	0.60	30	30	20	20
08	Con	0.59	0.54	0.64	27	32	23	18
09	Reg	0.59	0.38	0.80	19	40	31	10
10	Reg	0.68	0.56	0.80	28	40	22	10
	mean	0.61	0.53	0.69	26.6	34.4	23.7	15.3
	SD	0.06	0.07	0.09	3.66	4.30	3.77	4.30

**Table 5:** Comparison of implant type prediction performance based on CT-only morphology.

Tester	Input	Accuracy	A Se	R Se
Original Surgeons	Multimodal (full context)	100% (implicit)	N/A	N/A
Tester Surgeons	3D CT surface only	∼61%	53%	69%
ArthroNet^+^	3D CT surface only	∼78%	64%	92%

**Table 6:** Comparison with representative AI-based approaches in shoulder arthroplasty planning.

Study	Input Data	Task	Scope
2023 [30]	Clinical + imaging	Outcome prediction	ML-based decision support
2023 [31]	Clinical + CT planning	Implant selection	ML-assisted recommendation vs surgeons
2022 [24]	CT imaging	Implant sizing	3D preoperative templating
2021 [20]	CT imaging	Muscle degeneration	Soft-tissue assessment
2023 Geng2023	X-ray imaging	Implant identification	Postoperative recognition
**This work**	CT only	Implant type prediction	Morphology-driven analysis

## Data Availability

The dataset presented in this article is not publicly available. Requests to access the dataset should be directed to pietro.cerveri@polimi.it.

## References

[1] Kellgren J.H., Lawrence J.S. (1957). Radiological assessment of osteo-arthrosis. Ann. Rheum. Dis..

[2] Ogawa K., Yoshida A., Ikegami H. (2006). Osteoarthritis in shoulders with traumatic anterior instability: Preoperative survey using radiography and computed tomography. J. Shoulder Elb. Surg..

[3] Dallalana R.J., McMahon R.A., East B., Geraghty L. (2016). Accuracy of patient-specific instrumentation in anatomic and reverse total shoulder arthroplasty. Int. J. Shoulder Surg..

[4] Bartolotta R.J., Ha A.S. (2022). Current Imaging Concepts in Shoulder and Hip Arthroplasty. Radiol. Clin. N. Am..

[5] Horneff J.G., Serra López V.M. (2022). Preoperative Planning for Anatomic Total Shoulder Arthroplasty. J. Am. Acad. Orthop. Surg..

[6] Buck F.M., Jost B., Hodler J. (2008). Shoulder arthroplasty. Eur. Radiol..

[7] Bohonos C.J., Russell S.P., Morrissey D.I. (2021). CT versus MRI planning for reverse geometry total shoulder arthroplasty. J. Orthop..

[8] Elsharkawi M., Cakir B., Reichel H., Kappe T. (2013). Reliability of radiologic glenohumeral osteoarthritis classifications. J. Shoulder Elb. Surg..

[9] Mattei L., Pellegrino P., Calò M., Bistolfi A., Castoldi F. (2016). Patient specific instrumentation in total knee arthroplasty: A state of the art. Ann. Transl. Med..

[10] Villatte G., Muller A.S., Pereira B., Mulliez A., Reilly P., Emery R. (2018). Use of Patient-Specific Instrumentation (PSI) for glenoid component positioning in shoulder arthroplasty. A systematic review and meta-analysis. PLoS ONE.

[11] Levins J.G., Kukreja M., Paxton E.S., Green A. (2021). Computer-Assisted Preoperative Planning and Patient-Specific Instrumentation for Glenoid Implants in Shoulder Arthroplasty. JBJS Rev..

[12] Rojas J.T., Jost B., Hertel R., Zipeto C., Van Rooij F., Zumstein M.A. (2022). Patient-specific instrumentation reduces deviations between planned and postosteotomy humeral retrotorsion and height in shoulder arthroplasty. J. Shoulder Elb. Surg..

[13] Isensee F., Jaeger P.F., Kohl S.A.A., Petersen J., Maier-Hein K.H. (2021). nnU-Net: A self-configuring method for deep learning-based biomedical image segmentation. Nat. Commun..

[14] Wang G., Han Y. (2021). Convolutional neural network for automatically segmenting magnetic resonance images of the shoulder joint. Comput. Methods Programs Biomed..

[15] Marzorati D., Sarti M., Mainardi L., Manzotti A., Cerveri P. (2020). Deep 3D Convolutional Networks to Segment Bones Affected by Severe Osteoarthritis in CT Scans for PSI-Based Knee Surgical Planning. IEEE Access.

[16] Marsilio L., Moglia A., Rossi M., Manzotti A., Mainardi L., Cerveri P. (2023). Combined Edge Loss UNet for Optimized Segmentation in Total Knee Arthroplasty Preoperative Planning. Bioengineering.

[17] Moglia A., Marsilio L., Rossi M., Pinelli M., Lettieri E., Mainardi L., Manzotti A., Cerveri P. (2024). Mixed Reality and Artificial Intelligence: A Holistic Approach to Multimodal Visualization and Extended Interaction in Knee Osteotomy. IEEE J. Transl. Eng. Health Med..

[18] Marsilio L., Marzorati D., Rossi M., Moglia A., Mainardi L., Manzotti A., Cerveri P. (2025). Cascade learning in multi-task encoder–decoder networks for concurrent bone segmentation and glenohumeral joint clinical assessment in shoulder CT scans. Artif. Intell. Med..

[19] Thomas K.A., Kidziński L., Halilaj E., Fleming S.L., Venkataraman G.R., Oei E.H.G., Gold G.E., Delp S.L. (2020). Automated Classification of Radiographic Knee Osteoarthritis Severity Using Deep Neural Networks. Radiol. Artif. Intell..

[20] Taghizadeh E., Truffer O., Becce F., Eminian S., Gidoin S., Terrier A., Farron A., Büchler P. (2021). Deep learning for the rapid automatic quantification and characterization of rotator cuff muscle degeneration from shoulder CT datasets. Eur. Radiol..

[21] Wang R., Chen S., Ji C., Fan J., Li Y. (2022). Boundary-aware context neural network for medical image segmentation. Med. Image Anal..

[22] Allen C., Kumar V., Elwell J., Overman S., Schoch B.S., Aibinder W., Parsons M., Watling J., Ko J.K., Gobbato B. (2024). Evaluating the fairness and accuracy of machine learning–based predictions of clinical outcomes after anatomic and reverse total shoulder arthroplasty. J. Shoulder Elb. Surg..

[23] Longo U.G., Marino M., Nicodemi G., Pisani M.G., Oeding J.F., Ley C., Papalia R., Samuelsson K. (2025). Artificial intelligence applications in the management of musculoskeletal disorders of the shoulder: A systematic review. J. Exp. Orthop..

[24] Freehill M.T., Weick J.W., Ponce B.A., Bedi A., Haas D., Ruffino B., Robbins C., Prete A.M., Costouros J.G., Warner J.J. (2022). Anatomic Total Shoulder Arthroplasty: Component Size Prediction with 3-Dimensional Pre-Operative Digital Planning. J. Shoulder Elb. Arthroplast..

[25] Gupta P., Haeberle H.S., Zimmer Z.R., Levine W.N., Williams R.J., Ramkumar P.N. (2023). Artificial intelligence-based applications in shoulder surgery leaves much to be desired: A systematic review. JSES Rev. Rep. Tech..

[26] Potty A.G., Potty A.S.R., Maffulli N., Blumenschein L.A., Ganta D., Mistovich R.J., Fuentes M., Denard P.J., Sethi P.M., Shah A.A. (2023). Approaching Artificial Intelligence in Orthopaedics: Predictive Analytics and Machine Learning to Prognosticate Arthroscopic Rotator Cuff Surgical Outcomes. J. Clin. Med..

[27] Habermeyer P., Magosch P., Weiß C., Hawi N., Lichtenberg S., Tauber M., Ipach B. (2017). Classification of humeral head pathomorphology in primary osteoarthritis: A radiographic and in vivo photographic analysis. J. Shoulder Elb. Surg..

[28] Kleim B.D., Hinz M., Geyer S., Scheiderer B., Imhoff A.B., Siebenlist S. (2022). A 3-Dimensional Classification for Degenerative Glenohumeral Arthritis Based on Humeroscapular Alignment. Orthop. J. Sport. Med..

[29] Ladd L.M., Crews M., Maertz N.A. (2021). Glenohumeral Joint Instability: A Review of Anatomy, Clinical Presentation, and Imaging. Clin. Sport. Med..

[30] Simmons C., DeGrasse J., Polakovic S., Aibinder W., Throckmorton T., Noerdlinger M., Papandrea R., Trenhaile S., Schoch B., Gobbato B. (2023). Initial clinical experience with a predictive clinical decision support tool for anatomic and reverse total shoulder arthroplasty. Eur. J. Orthop. Surg. Traumatol..

[31] Shukla D.R., Rebolledo B.J., Aleem A.W., Jacquot A., Werthel J.D., Villacis D., Urvoy M. (2023). Effect of machine learning prediction on surgical decision making for shoulder arthroplasty: A multi-surgeon study. J. Orthop. Exp. Innov..

[32] Geng E.A., Cho B.H., Valliani A.A., Arvind V., Patel A.V., Cho S.K., Kim J.S., Cagle P.J. (2023). Development of a machine learning algorithm to identify total and reverse shoulder arthroplasty implants from X-ray images. J. Orthop..

[33] von Schacky C.E., Wilhelm N.J., Schäfer V.S., Leonhardt Y., Gassert F.G., Foreman S.C., Gassert F.T., Jung M., Jungmann P.M., Russe M.F. (2021). Multitask Deep Learning for Segmentation and Classification of Primary Bone Tumors on Radiographs. Radiology.

